# Changes in the Acute-Phase Protein Concentrations and Activities of Some Enzymes in Pigs Following the Repair of Experimentally Induced Articular Cartilage Defects Using Two Types of Biocement Powder

**DOI:** 10.3390/ani9110931

**Published:** 2019-11-07

**Authors:** Csilla Tothova, Jaroslav Novotny, Oskar Nagy, Petra Hornakova, Zdenek Zert, Maros Varga, Lubomir Medvecky, Katarina Vdoviakova, Jan Danko, Eva Petrovova

**Affiliations:** 1 Clinic of Ruminants, University of Veterinary Medicine and Pharmacy in Kosice, Komenskeho 73, 041 81 Kosice, Slovak Republic; Oskar.Nagy@uvlf.sk; 2 Clinic of Swine, University of Veterinary Medicine and Pharmacy in Kosice, Komenskeho 73, 041 81 Kosice, Slovak Republic; Jaroslav.Novotny@uvlf.sk; 3 Clinic of Horses, University of Veterinary Medicine and Pharmacy in Kosice, Komenskeho 73, 041 81 Kosice, Slovak Republic; petra.hornakova@uvlf.sk (P.H.); Zdenek.Zert@uvlf.sk (Z.Z.); 4 Sport-arthro Centre, Privat Hospital Saca-Kosice, 040 15 Saca-Kosice, Slovak Republic; maros.varga@nemocnicasaca.sk; 5 Institute of Materials Research SAS in Kosice, Watsonova 47, 040 01 Kosice, Slovak Republic; lmedvecky@saske.sk; 6 Institute of Anatomy, University of Veterinary Medicine and Pharmacy in Kosice, Komenskeho 73, 041 81 Kosice, Slovak Republic; katarina.vdoviakova@uvlf.sk (K.V.); jan.danko@uvlf.sk (J.D.); Eva.Petrovova@uvlf.sk (E.P.)

**Keywords:** acute phase proteins, serum enzymes, pigs, inflammation, cartilage defect, biocement

## Abstract

**Simple Summary:**

Articular cartilage reconstruction is aimed at the restoration of damaged joint cartilage. The use of bone cement is one type of method applicable for this reconstruction. The potential use of repair techniques must be evaluated by pre-clinical and clinical studies in animal models, including the assessment of some biochemical parameters. Acute-phase proteins are a class of proteins whose concentrations increase in response to injury or inflammation. They may serve as useful biomarkers for the evaluation of post-operative complications, as well as to reflect the extent of surgical trauma. Information regarding their usefulness after cartilage reconstruction are still limited. Similarly, little is known about the response of the organism to various reconstruction techniques and various biomaterials used for the repair of defects. This paper provides important information about the changes in the concentrations of acute-phase proteins and the activity of serum enzymes in pigs within the first 30 days following the repair of experimentally induced articular cartilage defects using tetracalcium phosphate/nanomonetite cement powder (C cement) and cement powder containing aminoacids (CAK cement). Marked inflammatory responses with increased acute-phase proteins concentrations were observed following the reconstruction of articular cartilage defects using both types of biocement powder. The results suggest, that the tetracalcium phosphate/nanomonetite cement powder without amino acids would be more suitable for possible cartilage repair in the human population.

**Abstract:**

The objective of the study was to assess the usefulness of acute-phase proteins (APPs) and serum enzymes in the evaluation of post-operative state after cartilage reconstruction in an animal model (*Sus scrofa domesticus*). Fifteen clinically healthy female pigs were evaluated during the first 30 days after the repair of experimentally induced articular cartilage defects using two types of biocement powders. Animals were divided into groups according to the type of biocement powder used: CAK—with amino acids (*n* = 6), C—without amino acids (*n* = 6) and the control group (Ctr) was without biocement (*n* = 3). The concentrations of selected APPs—serum amyloid A (SAA), haptoglobin (Hp) and C-reactive protein (CRP), and the activities of some serum enzymes—creatine kinase (CK), alkaline phosphatase (AP), and lactate dehydrogenase (LD) were measured one day before the surgery and on days 7, 14, and 30 after the surgical intervention. The most significant changes during the evaluated period were observed in the concentrations of SAA (*p* < 0.001) and Hp (*p* < 0.001), with marked increase of values 7 days after surgery. There was a numerical, but not statistically significant, difference between CAK, C and Ctr groups (*p* > 0.05). Marked variations were observed also in the activities of the evaluated enzymes, with the most significant changes in the activity of AP in the CAK group (*p* < 0.001). Presented results suggest possible usefulness of some APPs and serum enzymes in the evaluation of post-operative inflammatory state after the reconstruction of articular cartilage defects.

## 1. Introduction

Articular cartilage damage belongs to frequently occurring defects in the human population that are characterised by limited spontaneous healing potential [1,2]. Articular cartilage is avascular, aneural and alymphatic tissue. Inability to heal cartilage is attributable to a few factors such as chondrocyte immobility, limited ability of mature chondrocytes to proliferate, and the avascularity of cartilage. The repair of cartilage will mostly result in production of dense connective tissue [3,4]. Therefore, there is a need to develop new methods and materials for cartilage repair, especially by tissue engineering using different cell sources, biocompatible matrix scaffolds, biodegradable materials, as well as bioactive bone cements for reconstruction medicine [5,6,7]. Biocompatible bone cements can be successfully used as bone defect fillers in orthopedic research. Several calcium phosphate-based cements and biphasic mixtures have been prepared in order to obtain a mineral component of bone tissue as similar as possible to biological apatites [7]. They have a variety of origins, with slight differences in their composition, preparation and application technique on the defect, mechanical toughness, curing time, as well as resorption properties. Research on improving these new cements is still underway. The potential use of these materials in human clinical medicine must be evaluated by pre-clinical and clinical studies using animal models for articular cartilage defects [8]. In cartilage research, sheep have become a commonly used in vivo experimental animal model, especially because of body weight and bone regeneration time comparable to humans, as well as the presence of long bones [9,10]. However, sheep have been reported to have a very variable articular cartilage thickness (between 0.4–1.7 mm), which may cause difficulties in the design of the studies [11,12]. According to some researchers, the joint size in pigs, limited capability for endogenous repair of chondral and osteochondral defects, and the thickness of porcine cartilage are more closely similar to humans [13,14,15]. Because of these anatomical and physiological similarities to humans, pigs might represent another suitable model for in vivo cartilage research studies. The repair process and response in the animal models should be evaluated not only by clinical examination, radiographic testing, histopathological, and immunohistochemical methods, but also by biochemical and serum biomarker assessments. To the author’s knowledge, the only study dealing with the evaluation of post-operative inflammatory state after cartilage repair by the measurement of acute phase protein concentrations was conducted recently by Tóthová et al. [16] in sheep. These proteins were found to be useful biomarkers of both individual animal and herd health status [17]. Their synthesis may be markedly altered as a response to various attacks against the integrity of the organism, including infections, inflammation or tissue damage [18]. Furthermore, changes in the concentrations of acute phase proteins correlate positively with the degree of tissue damage, and are related to the severity of pathological processes [19,20]. Some acute-phase proteins were described as useful markers for the evaluation of post-operative complications, as well as to reflect the extent of surgical trauma [21]. However, the information regarding the usefulness of acute phase proteins in the evaluation of inflammatory responses after cartilage reconstruction are still limited. Similarly, little is known about the response of the organism to various reconstruction techniques, as well as to various biomaterials used for the repair of cartilage defects. 

In human medicine, there are many other markers that can be measured in blood serum and would be helpful also in cartilage regeneration studies. These include serum enzymes such as alkaline phosphatase (AP) for bone repair, and creatine kinase (CK) or lactate dehydrogenase (LD) for muscle damage. These enzymes are composed of several different isoforms, which are associated with different cell types and tissues, but only a few produce enough enzymes to alter their serum activity [22]. Thus, changes in AP activity may be associated with bone metabolism and cartilage lesions, while CK and LD are associated with muscle injury [23,24]. However, there are scarce data regarding the alterations in their activity caused by the repair of cartilage defects. Therefore, the present study was designed to evaluate the changes in the concentrations of selected acute phase proteins and in the activity of selected serum enzymes in pigs within the first 30 days following the repair of experimentally induced articular cartilage defects using tetracalcium phosphate/nanomonetite cement powder (C cement) and cement powder containing aminoacids (CAK cement). Calcium phosphate biocement based on tetracalcium phosphate/monetite mixture are used as bone-filling cements in the filling and repairing of bone defects. In this study, we applied standard calcium phosphate cement (with above composition and Ca/P ratio equal 1.67) and new composite cement with addition of four major amino acids present in collagen (with ratio similar in collagen) for filling of osteochondral defects artificially created in the knee. 

## 2. Materials and Methods 

### 2.1. Cement Preparation

The tetracalcium phosphate/nanomonetite cement (TTCPMH) powder mixture (for C cement) was synthesized by the in situ reaction between tetracalcium phosphate (TTCP) and diluted solution of orthophosphoric acid (86%, analytical grade, Merck) in solution with water:ethanol = 1:4 in the planetary ball mill with agate balls (3 balls, diameter = 1 cm) and in a vessel for 30 min. 

**Figure 1:**
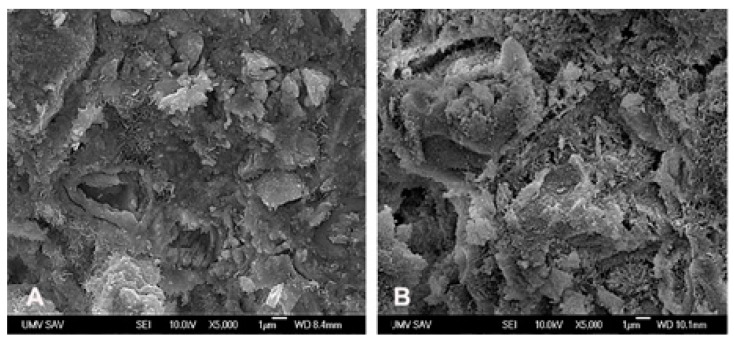
Microstructures of cements after hardening in simulated body fluid. (**A**) Irregularly shaped micropores with 1–5 µm size, larger agglomerates of 1–3 µm size composed of fine submicrometric globular particles and thin plate-like hydroxyapatite particles up to 500 nm length in C cement; (**B**) bigger micropores up to 10 µm dimension as a result pulling out of coarser calcium phosphate agglomerates from microstructure in CAK cement.

In the case of cements containing aminoacids (for CAK cement), four aminocids [(L-proline:glycine:4-hydroxyproline:L-arginine = 23:41:23:14 (mass ratio)] were added to orthophosphoric acid solution before milling. Total content of aminoacids in cements was 4 wt %. The final Ca/P mole ratio in TTCPMH was equal to 1.67. Tetracalcium phosphate (Ca_4_(PO_4_)_2_O, TTCP) was prepared by the solid state synthesis of an equimolar mixture composed of calcium carbonate (CaCO_3_, analytical grade, Sigma-Aldrich) and dicalcium phosphate anhydrous (DCPA) (CaHPO_4_ (Ph.Eur.), Fluka) at 1450 °C for 5 h. The product was milled in a planetary ball mill (Fritsch, 730 rpm, ZrO_2_ balls and vessel) for 2 h and the phase purity was analyzed using X-ray powder diffraction analysis (XRD, Philips X Pert Pro, Almelo, The Netherlands). The cement pastes were prepared by mixing cement powder mixture with hardening liquid (2% NaH_2_PO_4_ solution; Figure 1).

### 2.2. Animals and Sample Collection

The protocol of the study was approved by the State Veterinary and Food Administration of the Slovak Republic (No. 4650/17-221). Fifteen clinically healthy female pigs of the crossbreed of Large White and Landrace were included in the study, originating from a breeding farm with good herd health management (PD Agro, Michalovce, Slovak Republic). The animals were at the age of 5 months and weighed 77.4 ± 1.7 kg at the arrival to the Clinic of Swine of the University of Veterinary Medicine and Pharmacy in Kosice (Slovak Republic) where they were acclimatized 30 days before the scheduled beginning of the experimental procedure. At the clinic, the pigs were housed in groups of two or three animals in a solid floor pen with straw bedding. After surgery, they were housed individually in pens. They were fed with a commercial feed mixture intended for this category of pigs with free access to drinking water. 

Before the inclusion into the study, the pigs were physically examined using standard clinical examination procedures. The assessment of the general health state of the animals was oriented to the evaluation of feed intake, behavior, respiratory rates, as well as measuring of body temperature. The pigs were assessed also for physical injuries such as tail damage, lameness or skin lesions. After surgical intervention, the health status of the animals was monitored daily until the end of the study, and the evaluation of general health state was completed by the observation of local signs of inflammation in the surgical wound (heat, swelling, pain, discharge). The grade of lameness was estimated according to the lameness scoring system (from no detectable lameness, through minor to major lameness) described by Main et al. [25].

Blood samples for biochemical analyses were obtained from *v. jugularis* externa one day before the surgical intervention and repeated 7, 14 and 30 days after surgery. Blood was collected into serum gel blood collection tubes with clotting activator, but without anticoagulants (Meus, Piove di Sacco, Italy). The blood samples were allowed to coagulate at room temperature, and then were centrifuged at 3000× g for 30 min to separate serum from the clot. The harvested serum was aliquoted into plastic tubes, which were stored at −20 °C until the assay. 

After the 1-month post-operative period, the process of cartilage healing in the pigs was further evaluated till the end of the 3rd month after surgery by X-ray, computed tomography, and magnetic resonance imaging examinations, histological, immunohistochemical methods, and molecular analyses as well. The results of these analyses will be subject of another study. 

### 2.3. Surgical Procedure

**Figure 2:**
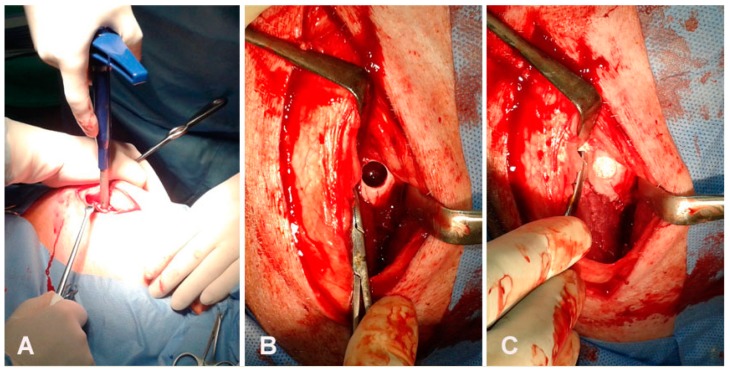
Surgical procedure. (**A**) Inducing of articular cartilage defect with osteochondral autograft transfer system (OATS) equipment, (**B**) Preparing articular cartilage defect in femoral trochlea before implantation, (**C**) Inserting of scaffold into prepared articular cartilage defect.

Feed and water in the pigs were withheld for 12 h before the surgery. General anesthesia was induced with a mixture of buthorphanol (0.1 mg/kg, Butomidor 10 mg/mL, Richter Pharma, Wels, Austria), azaperone 2 mg/kg (Stresnil 40 mg/mL, Janssen Pharmaceutica, Beerse, Belgium) administered intramuscularly, and ketamin 20 mg/kg (Ketamidor 100 mg/mL, Richter Pharma, Wels, Austria) administered intravenously. After anesthesia, a defect in the articular cartilage of the left stifle joint was created. The incision was conducted from the left lateral side, from medial patellar ligament distal to the tibial tuberosity. The stifle joint was visualized above the medial femoral condyle load. The subcutaneous tissue and superficial fascia were incised. After flexion of the stifle joint and partial subluxation of the knee, a defect on articular cartilage was made by the osteochondral autograft transfer system (OATS, Arthrex, Naples, FL, USA) at the distal epiphysis of the femur (trochlea) at a diameter of 8–10 mm and a depth of 8–12 mm. The site of the created defect was then filled with a biocement powder prepared according to Medvecky et al. [26]. According to the type of biocement used, the animals were divided into the following groups: CAK—biocement powder containing amino acids (*n* = 6); C—biocement powder without amino acids (*n* = 6). At body temperature, the biocement powder solidified within a few minutes, and the site of the defect was closed by standard surgical procedures (Figure 2). The same surgical technique was used to create a cartilage defect in the animals from the control (Ctr) group (*n* = 3), but without filling with biocement powder. All pigs received post-operative systemic broad spectrum antibiotic oxytetracyclinum dihydricum 20 mg/kg (Alamycin LA a.u.v., Norbrook, Newry, UK, once every second day) for 7 days and non-steroidal anti-inflammatory drug flunixin meglumine 2.2 mg/kg (Flunixin a.u.v., Norbrook, Newry, UK, once a day) administered intramuscularly for 7 days.

### 2.4. Laboratory Analyses 

Blood serum was evaluated for the concentrations of selected inflammatory markers—serum amyloid A (SAA, μg/mL), haptoglobin (Hp, mg/mL) and C-reactive protein (CRP, μg/mL), and for the enzyme activities of creatine kinase (CK, µkat/L), alkaline phosphatase (AP, µkat/L), and lactate dehydrogenase (LD, µkat/L). SAA was measured by sandwich enzyme-linked immunosorbent assay (ELISA) using commercial multispecies kits (TP-802, Tridelta Developmet, Kildare, Ireland) according to the manufacturer’s instructions. Porcine CRP was determined by solid-phase ELISA immunoassay using commercially available tests (TA-901, Tridelta Developmet, Kildare, Ireland). Haptoglobin was analyzed spectrophotometrically using commercial colorimetric kits (TP-801, Tridelta Development, Kildare, Ireland) in microplates, based on Hp-haemoglobin binding and preservation of the peroxidase activity of the bound haemoglobin at low pH. The absorbances were read on automatic microplate reader Opsys MR (The Dynex Technologies, Chantilly, VA, USA). The results were calculated using the computer software Revelation QuickLink version 4.25 (Dynex Technologies, Chantilly, VA, USA). The activities of serum CK, AP, and LD were assessed by commercial clinical chemistry assay kits (Randox, Crumlin, UK) on an automated biochemical analyser Alizé (Lisabio, Poully en Auxois, France). 

### 2.5. Statistical Analyses

The statistical analyses of the data were performed using the statistical software programme GraphPad Prism V5.02 (GraphPad Software Inc., San Diego, CA, USA). Arithmetic means (x) and standard deviations (SD) were determined by descriptive statistical methods for each evaluated variable and sample collection time. The distribution of data was evaluated by the Kolmogorov–Smirnov test for normality, which showed a non-parametric distribution of the most of the obtained results. Therefore, the non-parametric Friedman test was used to examine the changes in values during the post-operative period. The significance of differences in values between the different time points of sample collection was evaluated by Dunn’s multiple comparison post hoc test. For the analysis of the differences between the groups, the non-parametric Kruskal–Wallis test with Dunn’s post hoc test was used. 

## 3. Results

The surgical wounds developed no signs of inflammation and were without discharge in all the evaluated animals. The animals have shown improvement with no serious complications and inflammatory processes. 

The data obtained during the perioperative period are presented in Table 1. In all of the groups of animals significant changes in the concentrations of the inflammatory biomarkers during the evaluated period were found (*p* < 0.05–*p* < 0.001).

A trend of increasing concentrations 7 days post-surgery compared to the preoperative values with a subsequent decrease were observed for SAA, Hp, as well as CRP in all groups of animals, but the extent of increase was numerically different. While the concentrations of SAA in the CAK group increased more than 15-fold, its values in pigs from the group C increased only approximately 5 times and in control animals about 2.7 times. Similarly, Hp values in the CAK group increased approximately 7-fold, while in the C group we found a 2.5-fold and in control animals 2.2-fold increase. Compared to SAA and Hp, CRP showed only about a 1.6-fold increase by the use of both types of biocement powders, while in control animals the rise in values was only slight. Despite these marked numerical changes, the differences obtained post-surgically between the groups of animals were statistically not significant (*p* > 0.05). A trend of decreasing values was observed from the 14th post-operative day for all the evaluated acute phase proteins, reaching the preoperative or lower concentrations 1 month after surgery (*p* < 0.05–*p* < 0.001). Changes in the dynamics of the observed inflammatory proteins were less pronounced in the control group of pigs.

Significant changes in the dynamics of the values of all evaluated enzymes during the evaluated period were observed in the control group of pigs (*p* < 0.05 and *p* < 0.01). In AP, a significant dynamic was also found in the CAK group of pigs (*p* < 0.001). The activity of CK and LD in the CAK group increased non-significantly gradually till the end of the evaluated post-surgical period, showing approximately a 4-fold increase of CK activity 30 days post-surgery compared to the presurgical values. On the other hand, the activity of these enzymes in group C showed a non-significant increase of values 7 days after the surgery with a subsequent gradual decrease until day 30 post-surgery approximately to pre-operative values. More marked rise of values was recorded in the activity of CK. In the control group of pigs the activity of both enzymes showed similar tendency of changes with increase of their activity 7 days post-surgery, presurgical activity was approached 14 days after surgery and then a repeated increase of values was recorded 30 days after the surgical intervention. The changes in the activity were more marked in CK compared to LD. The activity of AP decreased 7 days post-surgery in both groups of experimental animals, as well as in control pigs with a subsequent gradual increase approximately to values that were recorded prior to surgery. The highest significance of changes was found in pigs of the group CAK. There was no significant difference in CK, AP and LD activities between CAK, C and Ctr groups (*p* > 0.05). 

## 4. Discussion

To evaluate successful cartilage repair and predict an uncomplicated healing process, the assessment of some parameters would be helpful. These should be easily and objectively measurable in blood serum, as well as correctly interpreted. The concentrations of acute-phase proteins increase in several diseases and inflammation of various origins, but their importance lies in the evaluation of their concentrations over time for monitoring of disease course, therapy, as well as post-operative period, and prognostic prediction also in surgical patients [27,28]. Furthermore, they may have important role in the detection of post-surgical complications [29], which might be useful also in the early identification of post-surgical complications and uncontrolled inflammatory processes after cartilage reconstruction. Some acute-phase proteins, which have been widely studied in human medicine as disease markers, might be one of the most useful tools that could be used to evaluate treatment response after cartilage reconstruction, as well as to determine the prognosis. In the current study, we evaluated the response of the organism in pigs by the assessment of selected inflammatory markers to the repair of induced cartilage defects using two types of biocement powder: tetracalcium phosphate/nanomonetite cement powder and cement powder containing aminoacids. Presented results showed significant alterations in all of the investigated acute-phase proteins with the highest concentrations 7 days after the induction of cartilage defects and their repair. However, the concentrations of the measured acute-phase proteins exhibited different response patterns with marked differences in the rate and magnitude of increase among the measured proteins, and marked numerical differences between the different animal groups. 

It has been shown in humans that the concentrations of C-reactive protein markedly increase after conventional total joint arthroplasty, and reflect the degree of systemic inflammatory response to surgical trauma [30,31,32]. Yamamoto et al. [33] reported that the evaluation of CRP could improve the assessment of post-operative inflammatory state and clinical decision making during recovery after surgery in dogs. Compared to the other inflammatory proteins, CRP as major acute-phase protein has been suggested to be a highly sensitive and reliable marker of systemic inflammation also in swine [34]. Our results showed only about a 1.6-fold increase by the use of both types of biocement powder, which suggest that CRP is not the best biomarker to assess the post-operative inflammatory state in pigs. Furthermore, the baseline values obtained for CRP in our study before the surgical intervention were higher than those reported in some previous studies [35,36]. One of the explanations for this may be the manipulation with the pigs before the intervention, their isolation and the withholding of feed and water before surgery, which might evoke stress in the evaluated animals and consequently the production of CRP in higher amounts. In our study, only a very slight increase of CRP concentrations was observed also in the group of control pigs with the creation of cartilage defects during the surgical intervention, but without filling with biocement powder. This increase may be attributed to the surgical procedure and operative trauma. 

More marked changes were observed in the concentrations of SAA and Hp, with the highest concentrations obtained on day 7 after the surgical intervention in both groups of experimental animals, but the extent and magnitude of changes was different depending on the measured protein, as well as type of biocement used for the reconstruction. While the concentrations of SAA in the CAK group increased more than 15-fold, its values obtained in pigs from the group C increased about 5 times. The concentrations of Hp in the CAK group showed approximately a 7-fold increase on day 7 after surgery, while in the C group its values increased about 2.5-times. These variations may reflect the differences in the reactivity of various acute-phase proteins to the damage and impaired homeostasis caused. While SAA was characterized by marked and rapid increase upon stimulation, Hp was defined as moderate acute-phase protein that usually has a slower and mild increase compared to SAA. Hp has an important function to bind myoglobin derived from traumatized muscle cells and hemoglobin from damaged erythrocytes to facilitate degradation and avoid oxidative stress caused by hemoglobin [37,38]. A trend of decreasing values was observed from the 14th post-operative day, reaching the preoperative concentrations 1 month after surgery, which suggests normal post-operative healing, the absence of inflammatory processes and restoration of homeostasis. On the other hand, persistently high or increasing concentrations of acute-phase proteins may indicate complications after surgery, ongoing inflammatory processes, infection of the surgical wound or lack of treatment response [27,39]. Similar findings were presented by Tóthová et al. [16] in sheep after the reconstruction of experimentally induced articular cartilage defects using polyhydroxybutyrate/chitosan-based biopolymer material, with significantly increased concentrations of Hp, SAA, and CRP after the surgical intervention and a subsequent decrease during the one-month post-operative period. Similarly, Aulin et al. [40] found in rabbits a pronounced increase of SAA concentrations after the surgical induction of full thickness osteochondral defect in the femorotibial joints and its decrease to preoperative values four weeks post-operatively. To our knowledge, no further studies were conducted to describe the inflammatory responses of the organism to the reconstruction of induced articular cartilage defects. The aforementioned differences in the dynamics of changes in the concentrations of evaluated acute phase proteins observed between the groups of animals might be caused by the degree of operative trauma, subsequent stress response, methods and invasiveness of surgical procedure, the level of surgeon experience, anesthetic protocol, course of regeneration and healing, antimicrobial and anti-inflammatory treatment during the post-operative period [41,42], as well as by the type and composition of biocement powder used, or the addition of amino acids into the cement powder. However, further studies are needed to explain these differences (although non-significant) obtained between the evaluated groups of pigs treated with different types of biocement powder, which would be important in deciding which biocement would be more suitable for possible human use. 

Marked alterations were observed also in the activities of evaluated serum enzymes, but similarly to inflammatory markers, the pattern of changes differed (although non-significantly) according to the biocement used. While the activity of CK, as well as LD in the CAK group increased gradually till day 30 post-surgery, the values obtained in the group C showed a more marked increase 7 days after surgery with a subsequent gradual decrease till the end of the evaluated post-operative period. The enzyme CK is found predominantly in skeletal muscle and its increased activity in serum is primarily associated with muscle damage, skeletal myopathy, and skeletal muscle necrosis. Furthermore, the changes in CK activity over time after an injury correlate well with the extent of muscle damage [43]. Marked changes in serum CK have been demonstrated also following muscle-cutting surgery, while the extent of alterations was related to the nature of procedure, extent of muscle involved, as well as to surgical incisions made [44]. A post-operative rise caused by muscle trauma and a subsequent decline of serum CK was observed also in dogs undergoing hemilaminectomy and ovariohysterectomy [28]. However, to our knowledge no previous study has examined the alterations in CK activity following cartilage repair, but it seems that muscle damage caused by the surgical procedure contributed to the considerable increase of its activity post-surgery. However, the factors affecting the extent of changes and the explanation of differences obtained between the two groups of experimental animals (according to the type of biocement used) should be further evaluated. Furthermore, CK is one of the serum enzymes that are most closely correlated with stress susceptibility, which may be present especially in stress-sensitive pigs resulting in markedly high serum CK activity [45]. This factor should be also taken into consideration by the interpretation of results. A very similar trend of changes was observed in the serum activity of LD following the cartilage repair, which similar to CK may be related to skeletal muscle damage and injury caused by the surgical intervention [46]. On the other hand, in cartilage tissue of horses, LD4 and LD5 were found the predominant isoforms, as they play an important role in anaerobic metabolic pathways [47]. Therefore, although often not routinely measured separately, it may be helpful to consider the different isoforms of this enzyme when interpreting the total activity [22]. Seeing that the most of studies dealing with the serum activities of enzymes and their isoform in various diseases and disorders of animals were performed many years ago, further studies would be helpful to better describe their changes also following cartilage repair. 

In the activity of AP, a more marked decline of values was observed 7 days after the surgical intervention with a subsequent gradual increase approximately to values obtained prior to surgery. Alkaline phosphatase exists as several isoenzymes with many different functions in the organism (corresponding to intestinal, placental, liver/bone/kidney) [48]. Because of its high expression in bones, AP is the most frequently used biomarker for the detection of active osteoblastic bone formation and osteogenic activity [49]. Thus, in tissue engineering, robust AP expression suggests successful osteogenesis [50]. On the other hand, the expression of AP has been demonstrated also in cartilage tissues [51]. Therefore, the alterations in its activity observed in our study may be attributed to these functions. However, further studies using electrophoretic separation or by the direct measurement of bone-specific alkaline phosphatase would be helpful in order to determine if this isoform is responsible for the aforementioned alterations. 

## 5. Conclusions

In conclusion, the results presented in this study suggest marked inflammatory responses of the organism following the reconstruction of induced articular cartilage defects, characterized by the increase of evaluated acute-phase proteins and changes in enzyme activities after the surgical intervention. The extent and dynamics of changes differed numerically, but non-significantly according to the type of biocement used. A trend of decreasing values from the 14th post-operative day was observed in both experimental groups, as well as in control animals, reaching the preoperative concentrations 1 month after surgery, which suggest normal post-operative healing. In this study, the use of biocement without amino acids showed better results and weaker inflammatory responses compared to the biocement with amino acids. The results suggest that the tetracalcium phosphate/nanomonetite cement powder without amino acids would be more suitable for possible cartilage repair in the human population. Because regenerative medicine is an intensively developing area of research, it would be of practical use and importance to identify laboratory biomarkers that could evaluate the post-operative inflammatory state following the reconstruction and repair of cartilage defects. Therefore, further studies are necessary before the possible implementation of the aforementioned inflammatory markers as routinely used diagnostic markers that could assess the post-operative period following the repair of articular cartilage defects. This study represents the first step towards achieving this goal in the future. The possible clinical application of the presented results is that the repeated measurement of selected inflammatory markers, together with clinical investigation of the patient, could be useful in the assessment of inflammatory state in the post-operative period and evaluation of the course of repair processes after cartilage reconstruction. 

## Figures and Tables

**Table 1:** Changes in the concentrations of serum amyloid A (SAA), haptoglobin (Hp) and C-reactive protein (CRP), and in the activity of creatine kinase (CK), alkaline phosphatase (AP) and lactate dehydrogenase (LD) in different groups of pigs 1 day before the surgical intervention and in the evaluated post-operative period (mean ± standard deviation (SD)).

Variable	Group of Animals	Sample Collection				*p*-Value
		Before Surgical Intervention	7 Days after Surgical Intervention	14 Days after Surgical Intervention	30 Days after Surgical Intervention	
SAA (µg/mL)	CAK	9.55 ± 14.58	152.60 ± 223.2 ^A^	50.46 ± 98.48	3.51 ± 5.33 ^A^	<0.001
	C	11.05 ± 17.72	61.29 ± 51.67 ^A^	19.24 ± 21.20	4.35 ± 5.71 ^A^	<0.001
	Ctr	13.84 ± 7.25	38.01 ± 26.81	2.02 ± 1.47	3.23 ± 4.83	<0.05
Hp (mg/mL)	CAK	0.65 ± 0.46 ^a,†^	4.82 ± 4.05 ^a,A^	2.52 ± 2.51	0.58 ± 0.47 ^A^	<0.001
	C	1.25 ± 0.87 ^a^	3.24 ± 1.04 ^a,1^	1.83 ± 0.57	0.56 ± 0.44 ^1^	<0.001
	Ctr	1.82 ± 0.22 ^†^	3.98 ± 2.11 ^a^	2.06 ± 0.90	1.08 ± 0.53 ^a^	<0.05
CRP (µg/mL)	CAK	145.9 ± 96.3	234.0 ± 26.1 ^a^	188.5 ± 79.7	178.2 ± 69.3 ^a^	<0.01
	C	129.4 ± 72.6	208.7 ± 9.2 ^a^	152.5 ± 63.6	100.8 ± 72.7 ^a^	<0.05
	Ctr	208.0 ± 15.5	217.3 ± 6.1	139.9 ± 81.3	103.6 ± 77.2	<0.05
CK (µkat/L)	CAK	27.2 ± 17.4	55.9 ± 29.7	89.2 ± 96.9	101.1 ± 86.6	n.s.
	C	19.5 ± 8.2	72.1 ± 53.8	41.2 ± 38.9	24.0 ± 11.0	n.s.
	Ctr	15.3 ±7.0	99.3 ± 16.6	13.3 ± 8.9	44.4 ± 38.1	<0.05
AP (µkat/L)	CAK	2.67 ± 0.53 ^a,†^	1.46 ± 0.59 ^a,b^	2.12 ± 0.86	2.56 ± 0.24 ^b^	<0.001
	C	2.17 ± 0.46	1.49 ± 0.60	2.38 ± 0.77	2.31 ± 0.86	n.s.
	Ctr	1.42 ± 0.24 ^†^	1.02 ± 0.02 ^a^	1.63 ± 0.40	1.94 ± 0.15 ^a^	<0.05
LD (µkat/L)	CAK	12.7 ± 4.3	13.5 ± 2.8	14.9 ± 5.4	15.7 ± 8.3	n.s.
	C	11.2 ± 3.9	17.9 ± 8.8	15.2 ± 8.2	11.8 ± 3.0	n.s.
	Ctr	10.4 ± 1.4	23.1 ± 3.7	10.4 ± 0.5	15.4 ± 4.1	<0.01

*p*—significance of the Friedman test. The same superscripts in rows mean statistically significant differences between the sample collections (Dunn test, a,b: *p* < 0.05, A: *p* < 0.01, 1: *p* < 0.001), the superscripts in the same columns mean statistically significant differences between the groups (†: *p* < 0.05).
